# The Effect of Polyacrylic Acid and Sodium Trimetaphosphate on Dentin Hybrid Layer Remineralization: An *In Vitro* Study

**DOI:** 10.4317/jced.59574

**Published:** 2022-10-01

**Authors:** Ahmad-Fawzi Ali, Ahmed-Fawzy Abo-Elezz, Rehab-Khalil Safy

**Affiliations:** 1 Department of Conservative Dentistry, Faculty of Dentistry, Suez Canal University, Egypt

## Abstract

**Background:**

Adhesive monomers are not able to fully encapsulate collagen fibrils in hybrid layer leaving them vulnerable to time-dependent hydrolytic degradation. The current in vitro study was designed for investigation of the remineralization of the resin-dentin hybrid layer using biomimetic analogs by nanoleakage investigation.

**Material and Methods:**

Firstly, occlusal enamel of thirty human molars was removed exposing flat surface of dentin, then randomly divided into three main groups according to the different remineralizing protocols (n=10) (R): control group (R0), STMP group (R1), and biomimetic remineralizing group (R2). The dentin surface of the STMP and biomimetic remineralizing groups (R1 and R2) was treated with STMP solution, followed by self-etch adhesive application on dentin surface of all groups, and restored with double 2-mm thick layers of resin composite. Each tooth was sectioned perpendicularly to the resin-dentin interface for producing 1-mm thick slaps. Each group was subdivided into four subgroups according to the incubation time to 24 hours, one month, three months, and 4 months. Retrieved slabs were prepared for nanoleakage for evaluation of metallic silver particles distribution percentage at the resin-dentin interface using digital image analysis software.

**Results:**

There was a statistically significant increase in nanoleakage over time in all three groups. However, the third group showed the least increase in metallic silver uptake over time.

**Conclusions:**

Hybrid layer could be remineralized by using dual-biomimetic analogs (PAA and STMP).

** Key words:**Hybrid layer, remineralization, nanoleakage, polyacrylic acid, sodium trimetaphosphate.

## Introduction

The hybrid layer stability is unintentionally compromised during the procedure of resin and dentin bonding (1). After etching and rinsing, adhesive resin monomers cannot completely circumscribe the exposed collagen matrix, leaving them completely or incompletely exposed at the hybrid layer’s base, without the polymerized resin protection, that result in time-dependent hydrolytic degradation, and finally, the hybrid layer’s destruction (2). Limited durability of resin-dentin bond is the most challenging problem (3) caused by collagen fibrils degeneration and hydrophilic resin hydrolysis (4). The weakened hybrid layer causes collagen fibers nano-space leakage, subsequently restoration failure (5). The demineralized dentin zone is generated by inconsistency between demineralization and the resin infiltration depth, consisting of exposed collagen fibrils encircled by nanometer-sized and water-filled zones (6). Thus, these zones permit the uptake of silver nitrate, a phenomenon termed nanoleakage (7).

So, numerous strategies have been achieved for resin-dentin bond stabilization to prevent failure of restoration (8). Biomimetic remineralization is a modern way to enhance the resin–dentin interface after being formed for backfilling the demineralized dentin collagen with liquid-like amorphous calcium-phosphate (ACP) nanoprecursers by using biomimetic analogs of noncollagenous proteins (NCPs) (9,10), which enhance binding of amorphous calcium phosphate nanoprecursors to dentin collagen. Biomimetic remineralization depends on calcium-hydroxide release from the set portland cement interaction with phosphate-containing fluids (11).

Several techniques and materials were used for biomimetic remineralization using the noncollagenous proteins analogs such as sodium trimetaphosphate (STMP), a polyphosphate-containing biomimetic analog applied as a templating biomimetic analog of matrix phosphoproteins (12). Additionally, Polyacrylic acid (PAA) is applied as an analog to sequester calcium ions and stop ACP nanoparticles from being aggregated into larger particles before entering the collagen fibrils intrafibrillar water compartments (13).

Therefore, current study aimed to assess the effect of polyacrylic acid and sodium trimetaphosphate on dentin hybrid layer remineralization using nanoleakage investigation to evaluate microporous zones within the hybrid layer instead of using Rhodamine B uptake by Confocal laser scanning microscopy (CLSM), which used by several studies 14, due to its limited availability and accessibility. The tested null hypothesis in the current study was that PAA and STMP had no significant effect on resin-dentin hybrid layer nanoleakage.

## Material and Methods

Materials’ descriptions, composition, manufacturers, and batch numbers are described in (Table 1).

**Table 1:**
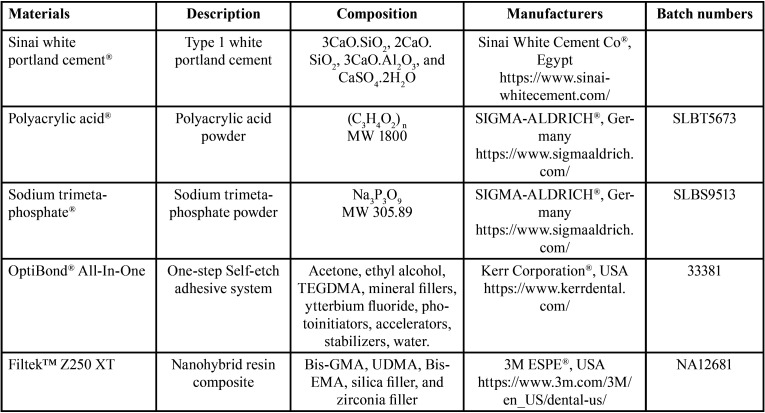
Materials’ description, composition, manufacturers, and batch numbers.

-Specimens’ preparation:

Thirty intact, sound, and freshly extracted human molars for periodontal reasons from patients aged 21- 40 years old were collected after the approval of the Research Ethical Committee of Faculty of Dentistry, Suez Canal University, Egypt (number 103/2018). Then, teeth were washed under running water, scaled to remove calculus, and polished with fine pumice and soft rubber cups at slow speed (14,15).

Each tooth was mounted in an acrylic resin block. A flat surface of dentin was exposed by removing the occlusal enamel and reaching the depth of 1 mm below dentin-enamel junction (DEJ) using the diamond saw (Isomet 4000, Buehler Ltd., Lake Bluff, United States) (16,17). Confirmation of the absence of enamel was done using a stereomicroscope at 10× magnification followed by dentin surface polishing with a slow-speed silicon carbide paper (14).

Then, the specimens were randomly divided into three main groups (R) according to the dentin pretreatment (n=10 each) using a randomization website (www.randomization.org) where, no dentin pretreatment was performed for the specimens of the control group (R0). Meanwhile, the specimens’ dentin surfaces of the second (R1) and third groups (R2) were pretreated with 2.5 wt% STMP that was hydrolyzed at pH 12 for five hours, then neutralized to pH 7.4 before usage (18). Each specimen’ dentin surface of the previously descried two groups was treated by the STMP for five minutes then allowed to dry.

One-step self-etch adhesive system (OptiBond All-In-One, Kerr) was applied following the manufacturer’s instructions on the dentin surfaces of all specimens, then polymerized for 10 seconds with the LED curing device (output intensity 1200 mW/cm2, Elipar B10, 3M ESPE, St Paul, MN, USA). Each specimen was encircled with a Tofflemire matrix band (Produits Dentaires SA, Vevey, Switzerland), followed by incremental application of double 2-mm-thick layers of Filtek Z250XT nanohybrid resin composite (3M ESPE, St Paul, MN, USA). Each increment was light-cured for 10 sec in accordance with the instructions of the manufacturer (14).

After that, each specimen was mounted on the automated diamond saw to be sectioned perpendicular to the resin-dentin interface for producing 1 mm thick resin-dentin slabs. Then, the four central slabs of each specimen were selected to be evaluated, so a total of 40 slabs was prepared for each group. The accuracy of the resin-dentin slabs thickness was checked using a digital caliper (ABSOLUTE Super Caliper SERIES 500, Mitutoyo, Japan).

-Remineralization procedures:

For the control group (R0), simulated body fluid (SBF) solution was prepared by dissolving 8.038 g/L NaCl, 0.355 g/L NaHCO3, 0.225 g/L KCl, 0.231 g/L K2HPO4•3H2O, 0.311 g/L MgCl2•6H2O, 0.292 g/L CaCl2, and 0.072 g /L Na2SO4 in deionized water, and adding 3.08 mmol/L Sodium Azide to stop the bacterial growth. Tris base at 6.118 g/L and 39 ml/L HCl were used to buffer the SBF to a pH value of 7.4, followed by filtering the solution (19).

For both R1 and R2 groups, white portland cement blocks were prepared by mixing type I white portland cement (Sinai white portland cement, Egypt) with deionized water in a water/powder ratio of 0.35:1 4, placed in flexible molds to set and age for one week at 100% relative humidity (20). Slabs were placed over the set white portland cement blocks inside glass vial.

The biomimetic remineralizing medium (BRM) was prepared by adding 500 μg/ml of PAA (molecular weight (MW) 1800; Sigma-Aldrich, St. Louis, MO, USA) to the SBF, with the pH of the latter adjusted to 7.4 (14).

Then, the glass vials containing the slabs were either filled with 15 ml of the SBF (R0 and R1) or BRM (R2), and stored at 37°C (21). Each group was further divided into four subgroups in accordance with storage time to 24 hours, one month, three months, and four months. Then, all glass vials were incubated at 37°C (Series B Classic Line, BINDER, Germany). The storage medium was changed every month, and the pH was monitored and adjusted weekly using a pH meter (Orion Star™ A111 pH Meter) to maintain it above 9.25 (14).

-Nanoleakage evaluation:

After each time interval, the corresponding resin-dentin slabs were retrieved, washed with physiological saline, and dried. The slabs were painted with double nail varnish layers (KIKO Milano, Italy), leaving only free 1 mm at the resin-dentin interface, and then instantly immersed in the tracer solution (50 wt% AgNO3) at pH 9.5 for 24 hours in total darkness 22, then, slabs were removed, washed with water for five minutes, and placed in a photo-developing solution for eight hours under fluorescent light. Slabs were polished with increasing fineness SiC paper (600–1200 grit) followed by a polishing cloth with 0.05 mm alumina particle suspension and cleaned in deionized water with ultrasonic for 30 minutes.

Scanning electron microscope (SEM) (TM-3000TM, Hitachi High Technologies Corporation, Tokyo, Japan) at 20.00 kV was used to examine slabs. All photomicrographs were taken at magnifications 1000X to demonstrate the tooth-restoration interface and assess the nanoleakage. Specific area (height × width =21 × 250 µm) of resin-dentin interfaces of each image was selected for metallic silver particles distribution analysis using digital image software (ImageJ 1.60, Scion, and Frederick, MD, USA). Using the Otsu method, the selected areas were converted into binary images; thus, the silver area was identified as dark spots on a white background to calculate the silver penetration percentage (22).

-Statistical analysis

The data were collected and analyzed using IBM® SPSS® Released 2013 (SPSS Inc., Armonk, NY: IBM Corp, NY, USA) Statistics Version 22.0 for Windows. Quantitative data were described using mean and the standard deviation for parametric data after testing normality using the Shapiro–Wilk test. The significance of the obtained results was judged at *P* ≤ 0.05. Two-Way ANOVA test was performed on different remineralization materials and time intervals on the nanoleakage percent area (Table 2).

**Table 2:**
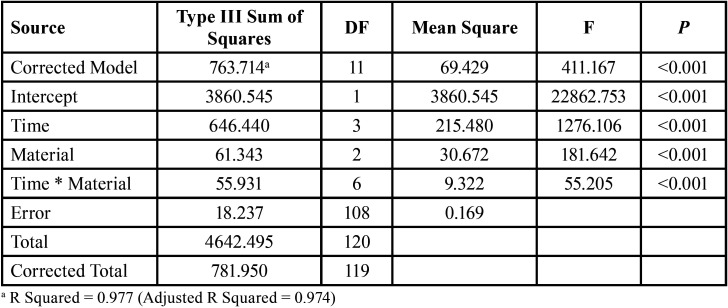
Two Way ANOVA test of time and material change (independent factors) on nanoleakage percent area (dependent).

## Results

**Figure 1:**
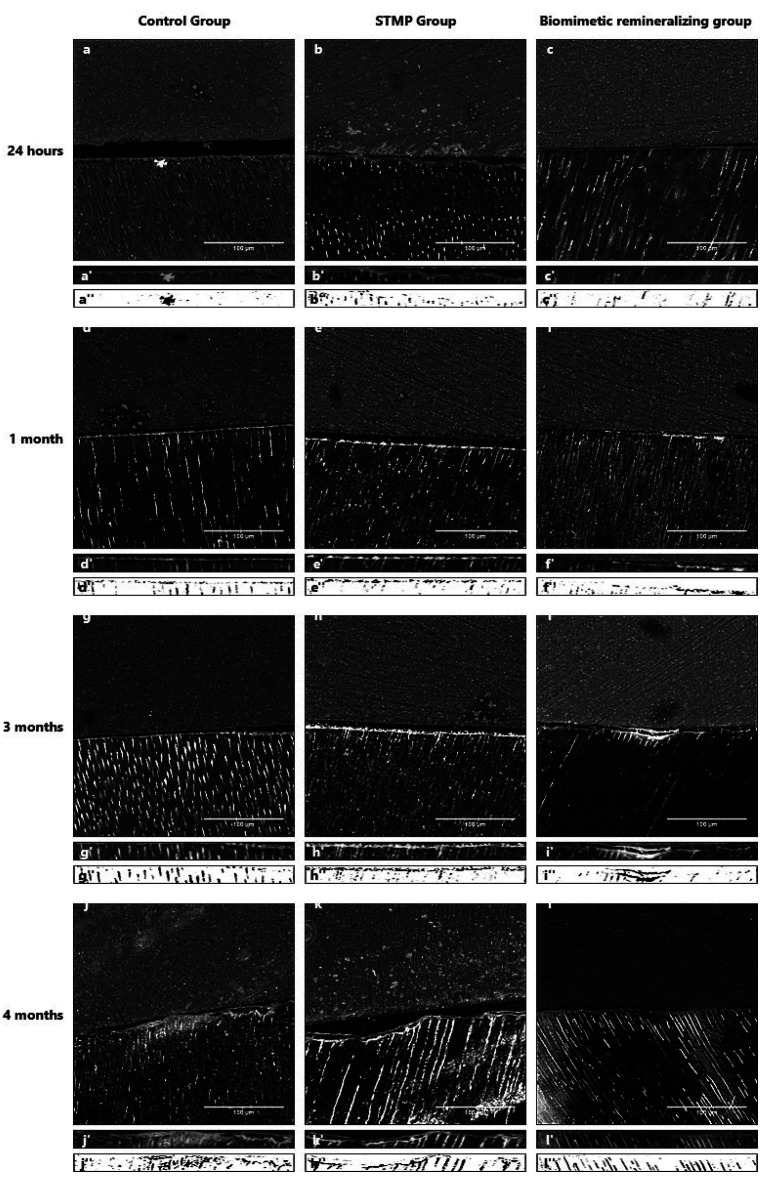
SEM photomicrographs of the resin-dentin hybrid layer’s nanoleakage at 1000X magnification (x), specifically selected area (x′), and the corresponding binary image (x′′).

**Figure 2:**
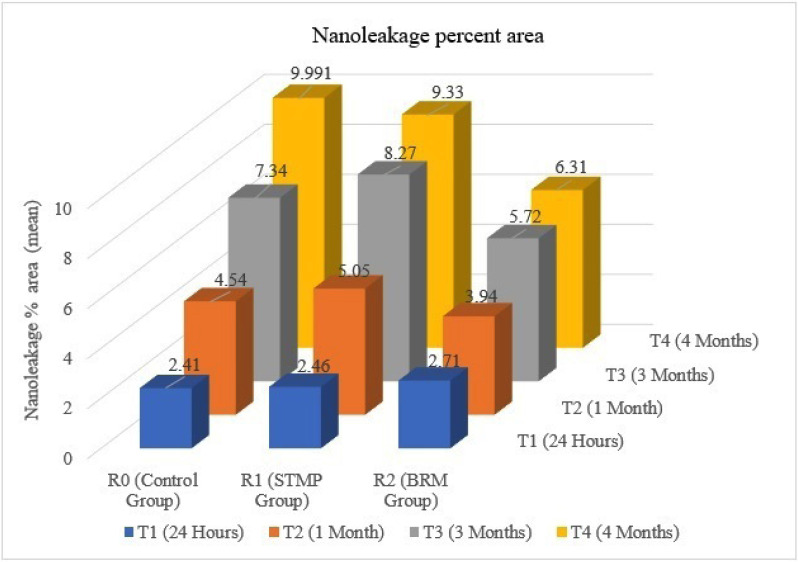
Bar chart showing mean nanoleakage percent area change between studied groups.

The results showed that the remineralizing material, time, and interaction significantly affected the nanoleakage percent area. The SEM photomicrographs and corresponding binary images of all tested groups showed that the silver particles deposition within the hybrid layer increased over time (Figs. 1,2).

SEM photomicrographs and corresponding binary images of the control group (R0) (Fig. 1) showed silver particles deposition within the hybrid layer was significantly increased in nanoleakage percent area over time from 24 hours to one month then to three months. The most significant mean value of nanoleakage percent area was at four months (Table 3).

**Table 3:**
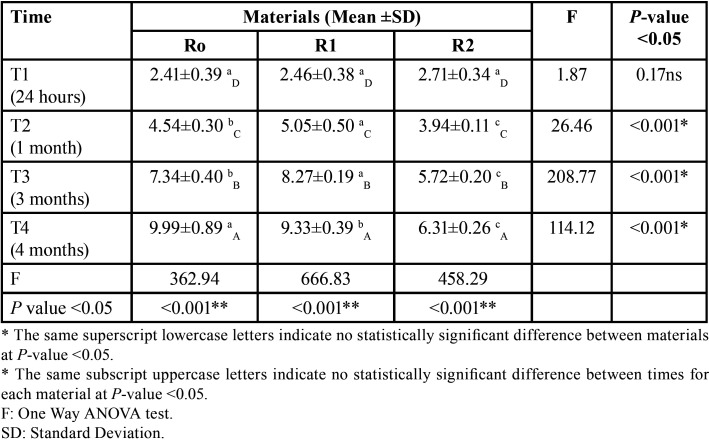
One Way ANOVA test comparing material change effect on the nanoleakage percent area.

Following the same pattern, the second group (R1) (Fig. 1) recorded a significant increase of nanoleakage percent area from 24 hours to one month then to three months with the most significant mean value of nanoleakage percent area was at four months (Table 3). Also, a significant increase of nanoleakage with the most significant mean value at four months followed by three months than one month with the least amount of nanoleakage was at 24 hours was recorded by the biomimetic remineralizing group (Fig. 1) (Table 3).

In comparing of the three tested groups, there was a significant increase of nanoleakage percent area from 24 hours to four months regardless of the remineralizing material. At 24 hours (T1), there was no significant difference in nanoleakage percent area between all three groups. After one and three months (T2 and T3), the third group (R2) showed the least nanoleakage percent area, followed by the R1 and R0 groups, respectively, with significant differences between them. After four months, R2 group showed the least nanoleakage percent area, followed by the control and R1 groups, respectively, with statistically significant differences between them.

## Discussion

The bond between resin and dentin depends on the adhesive system infiltration into the acid conditioned exposed collagen dentin matrix. The hybrid layer (HL) achieves a ‎basic role in the restoration’s micromechanical retention (10). Unfortunately, the adhesive ‎infiltration of collagen is incomplete due to its lower penetration capacity than the conditioned depth and difficulty of residual water removal in the dentin matrix (23). PAA is considered one of the most common noncollagenous proteins (NCP) analog, which simulates the ACP binding sites of dentin matrix protein 1 (DMP-1), so it plays an essential role in the dentin biomimetic mineralization and facilitating fluidic ACP nanoparticles to enter demineralized collagen fibrils (24).

On the other side, STMP could act ‎as a potential chemical phosphorylating agent to imitate matrix phosphoprotein-induced ‎remineralization since the phosphate groups can ‎be introduced onto type I collagen surface (25). The STMP in alkaline solutions is hydrolyzed into sodium tripolyphosphate by STMP ring-opening by hydroxyl group of sodium hydroxide (NaOH). The sodium tripolyphosphate has tribble phosphate groups, creating covalent bonding between the phosphate and hydroxyl group-containing amino acids on the collagen molecules side chains (26). Therefore, using PAA after pretreatment of collagen with sodium tripolyphosphate was shown to be a promising method of collagen remineralization. Consequently, this ‎ approach was referred as a dual-analog system, where the sodium tripolyphosphate act as a templating analog, resembling the more phosphorylated ‎domains of noncollagenous proteins (27).

It is worth mentioning that during the remineralization process, the minerals need to be in a certain way arrangement and small sized enough to be inserted into the collagen fibers’ gap zones. 14 Thus, nanoscopic pre-nucleation clusters outcome in quicker collagen fibril infiltration as a result, more rapid apatite formation is expected (28). The remineralization protocol that relay on portland cement, was launched in 2008 using biomimetic analogs for apatite remineralization guidance. 11 PAA with low molecular weight is used to imitate the dentin matrix protein 1 (DMP1) stabilizing function on ACP precursors, which are reduced to nanoscale by the interaction of portland cement with SBF (29).

SBF solution was freshly prepared at the start of each time interval and then incubated at 37oC for two days before use to ensure stability without precipitation (30) The biomimetic remineralizing medium was changed every month to ensure apatite formation instead of the unsTable octacalcium phosphate formation at a higher pH (14).

Evaluation of the HL remineralization in the present study was carried out using nanoleakage, which depends on assessing voids within the HL using ammoniacal silver nitrate uptake identified by the SEM 2 for up to 4 months as partial remineralization occurred at two months, and total remineralization of the dentin could be noticed in 90% after three to four months (11,31). Controlling regional differences in the current study was performed by utilizing only the central resin-dentin slabs from each specimen as the peripheral slabs may differ in dentin thickness (14).

According to the storage time, after 24 hours, the current study demonstrated that none of the tested groups was free from the nanoleakage, with no significant differences observed between them. This result might be due to incomplete resin infiltration into moist dentin throughout adhesive polymerization that provides water sorption channels as water can stay, infiltrate into the hybrid layer interface, or both and the silver deposition in the resin-dentin interface porous regions (5).

Additionally, aging from one month to four months, regardless of immersion medium, significantly increased nanoleakage percent area over time. This may be due to water absorption as the result of unprotected collagen network hydrolysis of incompletely resin-infiltrated hybrid layer, and collagen fibrils auto-degradation by dentinal matrix metalloproteinases and cysteine cathepsins (5).

The control and STMP groups showed significantly increased silver uptake over time in comparison to the biomimetic remonetizing group; this result could be explained on the basis of stabilization analog (PAA) absence in both of them that makes ACP microspheres to be too large to infiltrate the collagen fibrils’ internal compartments (31).

While the biomimetic remineralizing group based on using dual biomimetic analogs (STMP and PAA) showed significantly reduced uptake of silver particles than the control and STMP groups that could be allocated to the presence of PAA as a stabilizing agent with subsequent expected improved hybrid layer’s remineralization. This result might be due to the small-sized minerals, which were too small to be included in the empty zones of the collagen fibers and arranged in a specific way (14). This unique behavior allows partial remineralization within two months, and nearly complete remineralization could be observed after three to four months (11,31).

The current detection of the nanoleakage percent area assessments specified that the least amount of silver uptake within the HL was associated with using dual biomimetic remineralizing analogs in portland cement containing SBF that promotes the remineralization of the water-filled gapes inside the hybrid layer. Thus, the null hypothesis was rejected.

## Conclusions

Under the limitations of the current *in vitro* study, hybrid layer could be remineralized by using dual-biomimetic analogs (PAA and STMP). Storage for four months showed adverse effect on nanoleakage.
